# Calcite precipitation: The forgotten piece of lakes’ carbon cycle

**DOI:** 10.1126/sciadv.ado5924

**Published:** 2024-10-30

**Authors:** Gaël Many, Nicolas Escoffier, Pascal Perolo, Fabian Bärenbold, Damien Bouffard, Marie-Elodie Perga

**Affiliations:** ^1^ Institute of Earth Surface Dynamics, Faculty of Geosciences and Environment,University of Lausanne, Quartier Mouline, CH-1015 Lausanne, Switzerland.; ^2^ Surface Waters – Research and Management, Eawag, Swiss Federal Institute of Aquatic Science and Technology, Seestrasse 79, CH-6047 Kastanienbaum, Switzerland.

## Abstract

Lakes emit substantial amounts of carbon dioxide (CO_2_) into the atmosphere, but why they do remains debated. The long-standing vision of lakes as solely respirators of the organic matter leaking from the soils has been challenged by evidence that inorganic carbon produced by weathering of the catchment bedrock could also support lake CO_2_ emissions. How inorganic carbon inputs ultimately generate lake CO_2_ outgassing remains a blind spot. We develop and introduce a calcite module in a coupled one-dimensional physical-biogeochemical model that we use to simulate the carbon cycle of the large Lake Geneva over the past 40 years. We mechanistically demonstrate how the so-far neglected process of calcite precipitation boosts net CO_2_ emissions at the annual scale. Far from being anecdotal, we show that calcite precipitation could explain CO_2_ outgassing across various lakes globally, including some of the largest lakes in the world.

## Teaser

Calcite precipitation could explain CO_2_ outgassing across various lakes, including some of the largest lakes in the world.

## INTRODUCTION

Over the past three decades, the significance of inland waters as substantial greenhouse gas emitters has been repeatedly demonstrated (*1*, *2*), with carbon dioxide (CO_2_) released from these waters potentially offsetting up to 75% of the current terrestrial carbon sink (*3*). Nevertheless, important uncertainties persist regarding the root causes and processes behind CO_2_ supersaturation and subsequent net emissions, notably in lakes (*4*, *5*), limiting our ability to assess and forecast their variability in space and time.

In line with the conceptual view of the carbon cycle in soils, the carbon cycle of lakes has long been considered through an organic carbon lens. Lake heterotrophy, which causes respiration to exceed gross primary production chronically, is regarded as the primary cause of lake CO_2_ supersaturation (*2*, *6*, *7*). Despite being dominant, this metabolic paradigm fails to explain net CO_2_ emissions from autotrophic lakes (*4*, *8*). Besides, this organic-centered view is challenged by compelling evidence that hydrological inputs of dissolved inorganic carbon (DIC), rather than organic carbon, account for CO_2_ supersaturation in many lakes (*5*, *9*).

One blind spot in the inorganic carbon lens is the pathways by which DIC inputs from the lake catchments are converted into net CO_2_ emissions (*10*). The link appears straightforward whenever terrestrial DIC comes as direct inputs of CO_2_ inherited from soil respiration (*11*). Nevertheless, this situation is limited to some groundwater-fed lakes (*10*) and lakes in acidic catchments, as soil-derived CO_2_ evades quickly toward the atmosphere (*11*). Most of the hydrological inputs of DIC correspond instead to alkalinity [also called total alkalinity content (TAC), as mainly bicarbonate and carbonate ions]. TAC generated by carbonate and silicate catchment weathering (*12*) would sustain lacustrine CO_2_ emissions for up to half of the world’s surface occupied by lakes [e.g., hardwater lakes with TAC > 1 mol m^−3^, (*13*)]. Accordingly, internal lake processes are needed to link TAC inputs to surface CO_2_ supersaturation, on which literature has insofar remained elusive.

The carbonate equilibrium chemistry has been the default hypothesis: Assuming that the lake pH is lower than that of hydrological inputs, part of the catchment-inherited alkalinity could be converted into CO_2_ (i.e., H^+^ + HCO_3_^−^ ↔ CO_2_ + H_2_O). This is a reasonable assumption for boreal lakes (*5*) or heterotrophic lakes (*14*) whereby organic acids and respired CO_2_ maintain pH at, or below, circumneutral values. However, it is uncertain how much of the TAC can ultimately be turned into CO_2_ emissions by the sole carbonate equilibrium in lakes of moderate alkalinity where pH is comparatively higher and buffered. These lakes include some of the largest lakes in the world [such as Lakes Ontario, Erie, and Michigan, (*8*)].

At the same time, calcite precipitation (CP) is a common phenomenon in lakes (*15*). CP has been, for instance, reported in the Great Laurentian Lakes in the US (*16*), Great Lakes in Africa (*17*), and lakes in Northern China (*18*), South America (*19*), and Europe (*20*). The precipitation of 1 mol of calcite consumes 2 mol of alkalinity while producing 1 mol of CO_2_

$${\text{Ca}}^{2+}+{{\text{2HCO}}_{3}}^{-}↔{\text{CaCO}}_{3}+{\text{CO}}_{2}+H_{2}O$$

CP is closely linked to the primary production as photosynthesis sets the high pH conditions necessary for calcite supersaturation, while picoplankton cells (*20*–*22*), or macrophytes (*23*), often act as nucleation sites. CP has long been suspected of being pivotal in the carbon cycle of lakes, as annual CP fluxes can reach magnitudes similar to CO_2_ emissions (*10*, *23*, *24*). Nevertheless, there is an unresolved seasonal mismatch between the timing of CP, which occurs mainly during the warm, stratified season, and CO_2_ outgassing, which is maximal during mixing (*10*, *15*, *23*), so the role of CP on the lake’s CO_2_ emissions has been sidelined. Here, we aim to solve how and how much CP intervenes to transform catchment-inherited alkalinity as annual net CO_2_ emissions in lakes.

We first focus on Lake Geneva, a large (580 km^2^) and deep (309-m max depth), clear-water, and monomictic lake. Lake Geneva does not fit the metabolic paradigm as it is both autotrophic and a net CO_2_ source to the atmosphere (*4*, *25*). The waters are slightly alkaline (TAC ranging between 1.2 and 2 mol m^−3^). As a result of elevated water temperatures and high pH stemming from primary productivity, the surface layer of the lake becomes supersaturated with calcite, extending down to the lower boundary of the metalimnion in summer (*26*). CP occurs continuously throughout stratification (*26*), and the transformation of alkalinity into CO_2_ by CP fuels part of the aquatic primary production (*25*). Therefore, Lake Geneva provides an archetypal model site of an autotrophic lake whereby alkalinity inputs from its catchment could sustain net CO_2_ emissions via CP.

We aim to solve the seasonal and vertical dynamics of the entire carbon cycle of Lake Geneva and test whether, and eventually how, CP generates net CO_2_ emissions. We use a one-dimensional (1D) vertical modeling approach as an established method to inform on changes in the whole-lake seasonal dynamics (*27*). While carbon processes and fluxes also vary spatially, such a 1D model is not designed to evaluate short-timescale modulation due to lateral spatial variability. The modeled profile represents the seasonal dynamics with depth at an established reference point intended to inform whole-lake behavior. We simulate the lake’s organic and inorganic carbon dynamics by coupling a 1D physical model [SIMSTRAT, (*28*)] with a biogeochemical model [AED2, (*29*)]. As the CP and dissolution dynamics were not coded initially in AED2 (nor in any of the widely available lake C models), we have developed and integrated an additional module establishing the calcite equilibrium with respect to its saturation index, ΩCaCO_3_, based on field-based parametrization (see the Supplementary Materials) (*26*). The model is validated by in situ observations from the lake’s deepest point from 1981 to 2021, issued from the fortnightly monitoring (*30*). Then, we run simulations enabling or muting CP to quantify the share of CO_2_ flux due to the carbonate equilibrium only and to CP. In a second step, to validate the relevance of our approach, we extend the simulations of surface *P*co_2_ (partial pressure of CO_2_) to a broader range of alkalinity and compare simulations to observations of 18 other lake basins in Europe and the US, whereby CP had been documented.

## RESULTS

**Fig. 1.**
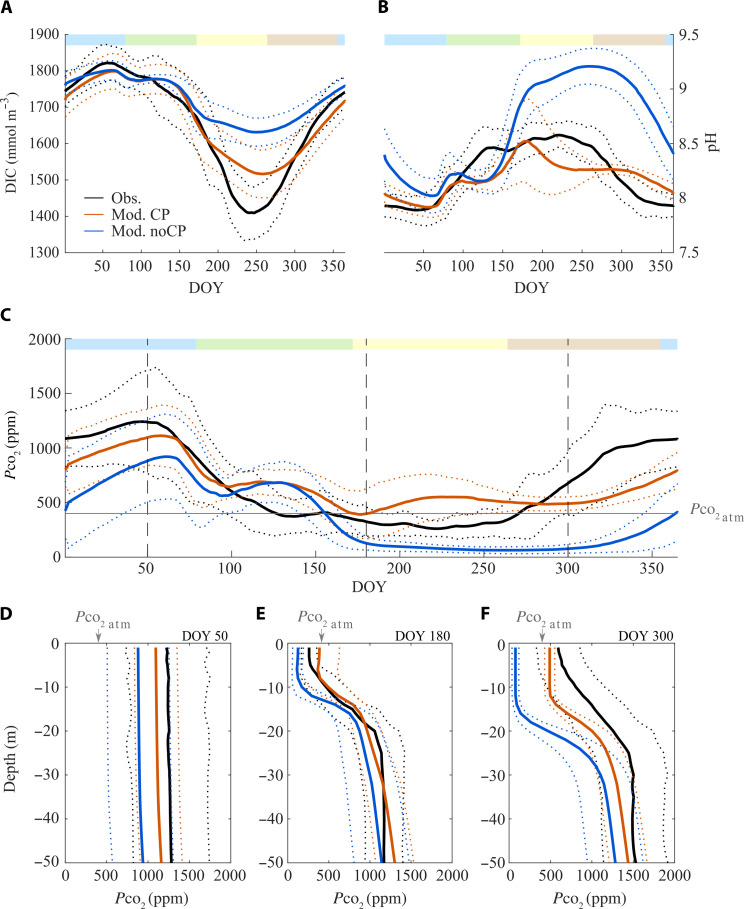
Climatology of observations and simulations. (**A** to **C**) Climatology of surface (averaged on 0- to 10-m depth) daily observations (Obs.) and models outputs when CP is enabled (i.e., the reference model, Mod. CP) or muted (Mod. noCP). DOY, Day of Year. The continuous colored line represents the daily average from 1981 to 2021; the dotted lines are the SD. The colored bar above represents seasons (sequentially winter-spring-summer-fall) (A) DIC, (B) pH, and (C) *P*co_2_. The gray line represents *P*co_2_ at equilibrium with the atmosphere. The vertical dotted lines indicate DOY 50, 180, and 300, for which the vertical *P*co_2_ profiles are exemplified in (**D** to **F**). Vertical profiles (daily average ± SD for 1981–2021) over the first 50 m of observed and simulated *P*co_2_ with enabled or muted CP at (D) DOY 50, (E) DOY 180, and (F) DOY 300. Color codes are the same for (A) to (C).

The seasonality of the variables related to the carbonate system in Lake Geneva over 1981–2021 is typical for a warm monomictic lake (Obs. in Fig. 1), with high DIC (1800 mmol m^−3^), minimal pH (<8), and elevated *P*co_2_ (~300% saturation) at the surface during winter mixing [Day of Year (DOY) 50; Fig. 1, A to D].

**Fig. 2.**
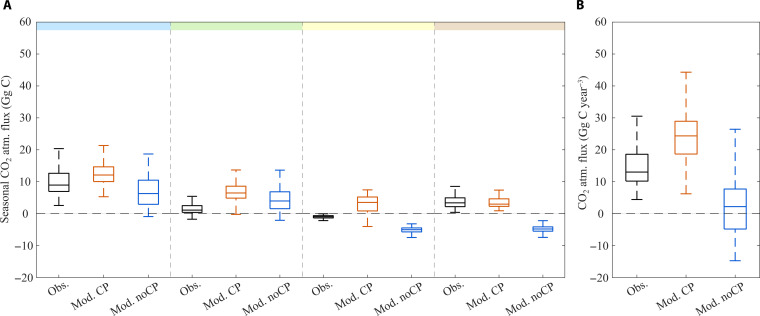
Comparison of the seasonal and annual CO_2_ atmospheric fluxes for observations (Obs.) and model outputs with enabled calcite precipitation (i.e., the reference model, Mod. CP) or muted calcite precipitation (Mod. noCP). Boxplots of (**A**) seasonal and (**B**) annual fluxes, presenting the median, interquartile, and non-outlier extremes for the 40 years of observations and simulations (1981–2021). The colored bar above represents seasons (sequentially winter-spring-summer-fall).

As the lake stratifies, the surface *P*co_2_ decreases due to diffusive emissions to the atmosphere (i.e., outgassing) and consumption by primary production, leading to an increase in pH (DOY 50 to 150; Fig. 1, A to C). DIC uptake by primary producers and CP also concurs to diminish surface DIC. In summer (DOY 150 to 250), surface DIC and *P*co_2_ reach their minimal values and maximum pH (~8.6; Fig. 1, A to C), leading to a slight undersaturation in CO_2_ as compared to the atmosphere [350 parts per million (ppm), i.e., ~85% saturation] yet limited to the first meters below the lake surface (<10 m; Fig. 1E). Convective mixing in the fall and winter (from DOY 250 onward) leads to thermocline erosion and deepening, bringing CO_2_ and DIC from the hypolimnion back to the surface while lowering the pH (Fig. 1, A to C and F). Over a complete annual cycle, the lake is a net C source to the atmosphere, with a median net CO_2_ outgassing of 12 Gg C year^−1^ (Fig. 2).

Our quantification of the role of CP on the carbon cycle relies on a fully validated model whose performances are detailed in figs. S3 and S4 and table S6. All the simulated variables show a good correlation with the observations made at the reference monitoring point [*r* > 0.6 and low root mean square errors (RMSEs)], except for nitrates and Particulate Organic Carbon (POC; *r* = 0.16 and 0.38, respectively; fig. S3). For all state variables of the carbonate system, i.e., surface DIC, TAC, and pH, simulations are in good agreement with observations (*r* > 0.8; Fig. 1, A and B) so that the model reproduces well the dynamics of surface *P*co_2_ (*r* = 0.8 and RMSE = 222 ppm; Fig. 1C and fig. S6). However, the climatology of surface dissolved oxygen and particulate organic carbon (fig. S6) highlights that the model underestimates primary production and CO_2_ consumption, especially in the early spring and late summer. Consistently, the modeled surface *P*co_2_ is, on average, 200 ppm higher during stratification than for observation data, leading to some overestimation of CO_2_ emissions at the annual scale. Despite slightly higher, modeled CO_2_ fluxes to the atmosphere are in the range of those computed from observation data (Mod. CP in Fig. 2).

In a modified version of the validated model (Mod. noCP), the CP and dissolution dynamics are muted to assess how CO_2_ fluxes change when only the carbonate equilibrium chemistry is considered. Surface *P*co_2_ differs between both scenarios more markedly in summer and fall, while differences are limited during the maximum of the winter overturn (Fig. 1, A to D). In the absence of CP, the DIC loss from the epilimnion during summer is only half that of the reference model (150 and 300 mmol m^−3^ for the model without and with CP, respectively; Fig. 1A). When CP is enabled (Fig. 1B, Mod. CP), pH values remain below 8.6 during summer, while, without CP, pH values rise well above 9 and remain high until the very end of the year (Fig. 1B, Mod. noCP). Reciprocally, while surface *P*co_2_ is close to the atmospheric equilibrium in summer and already supersaturated in fall in both the reference scenario and observations (Obs. and Mod. CP), surface *P*co_2_ is strongly undersaturated when the calcite dynamics is muted (100 ppm, 25% saturation; Fig. 1C, Mod. noCP). From the early summer (DOY 180 onward; Fig. 1E), *P*co_2_ is two to three times more depleted throughout the surface mixed layer (down to the 12-m depth) in the absence of CP. The CO_2_ depletion penetrates even deeper throughout summer (>20 m) so that the surface undersaturation persists longer and deeper throughout the fall in the absence of CP (DOY 300; Fig. 1F).

The gradient of partial concentrations primarily drives the diffusive fluxes at the lake-water interface. Consequently, variations in surface *P*co_2_ between the two versions of the model lead to clear variations in final CO_2_ emissions (Fig. 2). While CO_2_ emissions peak during the winter overturn, there are no substantial differences in emissions between actual observations and both scenarios at this season (Fig. 2A). In the observations and reference scenario, the lake is either neutral or a CO_2_ emitter to the atmosphere at all seasons (Fig. 2A), with net annual emissions ranging between 12 and 24 Gg CO_2_ year^−1^ (Fig. 2B). In contrast, in the absence of CP, the lake turns into a carbon sink not only during summer when the stratification is maximal but also during the fall, as the convective mixing does not yet compensate for the CO_2_ surface depletion inherited from the summer. As a result, without CP, the median value for the net CO_2_ emissions drops to 2 Gg C year^−1^, and the lake gets close to carbon neutrality toward the atmosphere at the annual scale. The difference in CO_2_ emissions with and without the calcite dynamics is close to 20 Gg C year^−1^, comparable to the averaged 17 Gg C of annually precipitated calcite as computed from the reference model.

## DISCUSSION

The inorganic carbon lens still struggles to find its way within the active pipe model (*2*) despite long-standing evidence of the role of hydrological inorganic carbon input on lakes’ C budgets (*5*, *9*, *10*). One potential reason might be our incomplete grasp of the processes that affect alkalinity (i.e., TAC) and DIC in lakes and how they affect CO_2_ exchanges at the lake-atmosphere interface (*10*). Considering that mechanistic models represent the available and consensual process knowledge at a given time, the fact that none of the carbon-centered lake models we know of includes the calcite dynamics illustrates this shortcoming.

We have used a lake biogeochemical model coupling organic and inorganic C processes to address how TAC inputs can support net CO_2_ degassing in an autotrophic lake. Models’ performances for lake biogeochemical variables are often more critical than physical variables (*31*). We have yet achieved a very high representativeness for the biogeochemical variables relevant to carbon dynamics. We acknowledge underestimating the primary production likely as a result of including only two functional phytoplankton groups (diatoms and green algae), which leads to an inherent simplification of the whole community dynamics. The accurate modeling of phytoplankton biomass is a recurrent issue of deterministic water quality models (*31*), and the parametrization of our model was a trade-off between accuracy and practicality (*32*). Here, the overall organic rates estimated in our simulations clearly underline the autotrophic character of the lake’s metabolism, with positive net ecosystem productivity values (NEP = 105 Gg C year^−1^) coherent with previous studies (*33*, *34*).

We set a constant precipitation flux once a threshold for calcite saturation is passed, while field data demonstrated that CP could be directly tied to primary production (*26*). The constant calcite flux corresponds to an average value derived from a highly resolved field parametrization (*26*) and was chosen to limit the addition of further uncertainties to those inherent to the modeling of primary production. The modeled CP, however, still depends on primary production, as the production level still sets the time when the calcite saturation threshold is reached but not the quantity of precipitated calcite. Despite the model simplification and modeling choices, the amount of calcite precipitated annually within the reference simulations is within values observed on the field (*4*, *26*). Overall, the model reproduces well the seasonality and vertical distribution of pH, DIC, and *P*co_2_ and meets the essential criteria for its use in studying the impact of CP on carbon fluxes. Further attempts may encounter similar difficulties in the representation of primary production. They may also resort to a parametrization with a fixed threshold for calcite saturation and a constant CP flux. However, since both are expected to vary from one lake to the other, increasing notably with the lake’s trophic status (*20*), we recommend adopting a site-specific calibration of those parameters.

The simulations show that when the carbonate equilibrium chemistry is the only mechanism by which DIC is converted into CO_2_, final CO_2_ emissions are close to carbon neutrality for Lake Geneva. Even for such an autotrophic lake with relatively buffered pH, the carbonate equilibrium can generate sufficient CO_2_ to compensate for the 105 Gg C of CO_2_ consumed by the net metabolism across the total water column. However, accounting for the CP is necessary to reach the annual supersaturation and a net CO_2_ outgassing observed for Lake Geneva.

The calcite dynamics first modifies the CO_2_ exchanges between the lake and the atmosphere during stratification when the surface *P*co_2_ is the lowest. The CO_2_ released by CP during stratification contributes to stabilizing the pH below 8.6 and buffers *P*co_2_ around 85% CO_2_ saturation throughout the mixed layer (instead of 25% CO_2_ saturation in the absence of CP). CP thus compensates partly for the CO_2_ loss due to primary production, maintaining surface *P*co_2_ close to saturation in summer. Therefore, CP reduces the amplitude of the *P*co_2_ gradient across the lake-atmosphere interface, preventing atmospheric CO_2_ invasion even in summer when the stratification and primary production are maximal. Besides, CO_2_ provided by CP constrains pH elevation, limiting the pH-driven chemical enhancement of fluxes (*35*). In contrast, without CP, surface CO_2_ depletion and high pH turn the lake into a sink of 6 Gg CO_2_ during summer.

Summer CP also affects the CO_2_ exchanges between the lake and the atmosphere during convective mixing (fall and winter). In summer, CP occurs down to the lowermost metalimnion depth, limiting CO_2_ undersaturation throughout the epilimnion. Surface *P*co_2_ then shifts back to oversaturation as soon as convective mixing, which brings back deeper CO_2_, starts with the fall cooling (Fig. 1F). In contrast, in the absence of CP, the CO_2_ undersaturation is severe down to the lower metalimnion. The convective mixing needs to reach much greater depths to restore supersaturation, and it is then ~100 days later, that the deep CO_2_ compensates for the summer loss (day 350; Fig. 1C). Without CP, CO_2_ invasion persists despite convective mixing in the fall, leading to an additional 5 Gg CO_2_ pumping. Together, CP acts on net CO_2_ exchanges by making the lake less of a C sink in summer and more of a C source during the fall convective mixing.

**Fig. 3.**
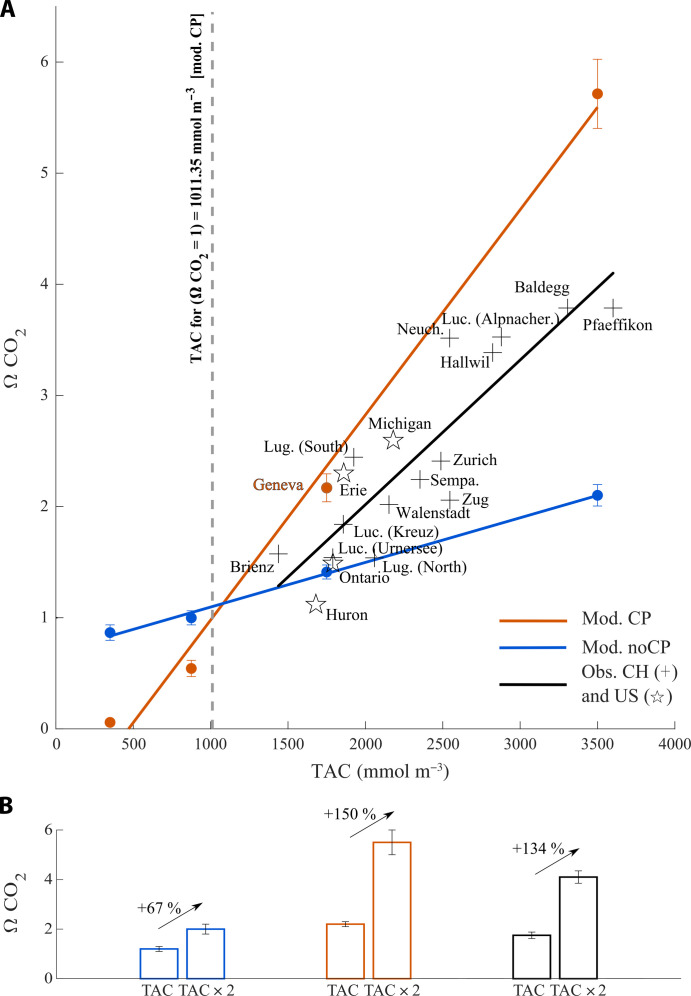
CO_2_ supersaturation as a function of alkalinity level. (**A**) Annual averages of CO_2_ saturation (ΩCO_2_) computed from the model’s outputs with (Mod. CP) or without CP (Mod. noCP) over a range of alkalinity representative of lakes whereby CP has been observed. The equations for the regression models are ΩCO_2_ = 1.84 TAC − 0.86 for the model with enabled CP (Mod. CP) and ΩCO_2_ = 0.42 TAC + 0.69 for the model without CP (Mod. noCP). Dots represent annual averages of CO_2_ saturation observed for lakes with CP. Cross-shaped symbols are for lakes in Switzerland other than Lake Geneva (data from the Swiss Federal Office for the Environment), and star-shaped symbols are for US lakes [data from (*38*)]. The black line represents the regression model for lake observations, with the following equation: ΩCO_2_ = 1.30 TAC − 0.60. (**B**) Changes in CO_2_ supersaturation due to a doubling of initial alkalinity (with Lake Geneva’s alkalinity as the reference) for, respectively, the model without CP, the reference model with CP, and the model fitted to the lakes’ observations.

The modeling exercise on Lake Geneva shows that CO_2_ outgassing is fueled by alkalinity but cannot be explained by the carbonate equilibrium alone. CP is the necessary mechanism by which the lake turns into a net CO_2_ emitter at the annual scale. To test our conclusions beyond the case of Lake Geneva, we ran additional simulations of surface *P*co_2_ with both iterations of the model, modulating the initial lake alkalinity for ¼, ½, equal to, and double that of Lake Geneva (TAC range: 0.4 to 3.6 mol m^−3^). Results are reported as annual averages of CO_2_ saturation (ΩCO_2_ $=\frac{{\text{pCO}}_{2}}{{\text{pCO}}_{2\;\text{eql}}}$), to which we fit a regression model (with or without CP) as a function of alkalinity (Fig. 3A).

All other conditions kept constant, annual ΩCO_2_ increases with the lake’s alkalinity for both model versions (Mod. noCP and Mod. CP). Even in the absence of CP, alkalinity above 1000 mmol m^−3^ would generate annual average CO_2_ supersaturation (Mod. noCP in Fig. 3A). Those results, in line with conclusions from US lakes (*14*) and Spanish reservoirs (*13*), corroborate that the sole carbonate equilibrium can turn enough alkalinity into net CO_2_ emissions for TAC > 1000 mmol m^−3^, even for autotrophic lakes. In the absence of CP, the model predicts that doubling alkalinity (using Lake Geneva as a reference) leads to a +67% increase in CO_2_ saturation (Fig. 3B) and lake CO_2_ emissions (assuming a constant piston velocity). However, the slope of the relationship between CO_2_ saturation and TAC is much steeper when CP is enabled (Fig. 3A). The same doubling of alkalinity boosts ΩCO_2_ and subsequent emissions by +150%, attesting that CP enhances the effect size of alkalinity on CO_2_ emissions.

We then superimpose to the extended model’s results the annual TAC and ΩCO_2_ obtained from 18 other lake basins (in the US and Europe), whereby CP had been reported (Fig. 3A). As for the modeled data, CO_2_ saturation in the observation dataset significantly increases along with lake alkalinity (*r*^2^ = 0.76, *P* < 10^−6^). The regression model fitted to the observation data predicts that a doubling of alkalinity leads to a +134% increase in CO_2_ saturation and lake CO_2_ emissions, i.e., an effect size similar to the one derived from the model with CP. Lakes from the dataset span an extensive range in size (surface areas vary over four orders of magnitude), trophic status (from oligo to eutrophic), and mixing regimes (from oligo to dimictic), all of which are likely to affect the inorganic carbon fluxes (table S8). However, the similarity in the effect size between the Mod. CP and the observational dataset suggests that both the model parametrization and the deduced mechanisms, i.e., CP boosting of CO_2_ emissions, apply broadly. Net CO_2_ outgassing observed especially for lakes of TAC ≥ 2000 mmol m^−3^, including some of the largest freshwater lakes in the world (i.e., lakes Erie and Michigan), does not solely result from the sole carbonate equilibrium and may be boosted by the conversion of alkalinity into CO_2_ outgassing through CP.

DIC accounts for two-thirds of the terrestrial carbon loadings entering surface waters and contributes half of the carbon that reaches the ocean (*36*, *37*). As for organic carbon, alkalinity is reactive and undergoes transformation along the hydrological continuum. Of the carbon lost by evasion or burial throughout transfer in inland waters, 72% is DIC (*36*). Notably, most (60%) of the increase in terrestrial carbon export from the land to the sea since 1880 arises from a greater DIC soil export due to climate warming and change in land use. Despite the evident significance of DIC in lateral carbon transport from land to sea, the active pipe concept has been implicitly reduced to the fate of organic carbon within inland waters (*2*, *38*, *39*). Our findings indicate that inorganic carbon should be reinstated to its rightful importance while considering aquatic carbon cycling. CP is a critical process for alkalinity loss in inland waters, which exclusion from carbon models substantially undermines the estimation of CO_2_ evasion, both presently and in future projections. Therefore, alkalinity and associated processes, such as CP, must be explicitly included in the active pipe model to ensure that inorganic carbon does not remain the forgotten yet dominant piece of the aquatic carbon cycle.

## MATERIALS AND METHODS

Lake Geneva is the largest hardwater lake in Western Europe, with a surface area of 580 km^2^. Lake Geneva has a mean water residence time of 11 years, and it defines part of the border between France and Switzerland at 372 m above sea level. Lake Geneva is an oligo-monomictic lake whose complete mixing happens every 7 years on average during exceptionally cold winters (*40*). The moderately alkaline waters of the lake (ranging between 1.2 and 2 mM) result from the weathering of carbonate rocks in the lake’s catchment. The Rhône River drains the Alps in the eastern part of the lake and represents the primary hydrological input (∼80%). DIC in the Rhône is 96% in the form of bicarbonates. Considering the pH of the lake waters (i.e., ∼7.9 to 9), bicarbonate ions make up 92% of the total lake C stock. CP, strongly coupled to primary production, removes 30 to 42 g C m^−2^ of TAC annually from the epilimnion (i.e., 17 to 25 Gg C considering the whole lake) (*26*). Annual CO_2_ emissions are ~10 to 20 Gg C year^−1^ (*27*). The lake NEP represents ~80 g C m^−2^ when accounting for the whole, autotrophic, and heterotrophic respiration between 0 and 30 m (*33*, *34*). Since 1957, Lake Geneva’s physical and biogeochemical conditions have been monitored by the Commission International pour la Protection des Eaux du Léman at the lake’s deepest point throughout the entire water column monthly or fortnightly (*30*).

We couple the 1D physical model SIMSTRAT (*28*) to the biogeochemical process model AED2 (*29*) to simulate the organic and inorganic carbon dynamics in Lake Geneva from 1981 to 2021 (see the Supplementary Materials). AED2 includes an inorganic carbon module that simulates the concentrations of all DIC species at all depths and time steps from (i) hydrological inputs to the lake and exchanges at the atmosphere and sediment interfaces, (ii) consumption and production through primary production and mineralization, and (iii) speciation through the carbonate equilibrium. Simulations of C processes through AED2 then require activating all available modules [temperature, dissolved oxygen, and nutrients (C, N, P, and Si) and the phyto-zooplankton dynamics]. The first simulations were run using this standard configuration. Despite successful calibration and validation for all state variables except carbon, simulated pH and DIC values were systematically off (either pH values were too high or DIC values too low). These offsets arose because CP and dissolution dynamics were not coded initially in AED2. On the basis of field-based parametrization of the relation between summer CP and NEP (*26*), an additional module has been developed and further integrated within the carbon module (see the Supplementary Materials). Briefly, this module calculates the equilibrium of calcite with respect to its saturation index, Ω_CaCO3_. CP is initiated whenever Ω_CaCO3_, calculated from the activities of the carbonate and calcium ions and calcite solubility constant, is >1.5. Beyond this threshold, CP is set at a constant rate of 2.5 mmol C m^−3^ day^−1^, consistent with observation data (*26*). Reproducing the calcium flux from the sediment imposes that all the authigenic calcite is redissolved in the sediment pore water.

Simulation outputs are generated at a 2-m-depth resolution and daily time step from 1981 to 2021. Once validated, the model is run for two experiments. Experiment 1 includes the full model accounting for calcite dynamics (reference model or Mod. CP). In contrast, in experiment 2, the calcite dynamics is muted (i.e., by fixing TAC to a constant value, Mod. noCP) to assess its effect on the variables of the carbonate system. Moreover, to evaluate the overall effect of TAC on the dynamics of CO_2_ supersaturation, we also run specific test cases varying the level of lake TAC, as detailed in the Supplementary Materials.

## Supplementary Materials

### This PDF file includes:

Supplementary Text Tables S1 to S8 Figs. S1 to S7 References
