# Supplementary material for: Calcite precipitation: The forgotten piece of lakes’ carbon cycle
Source: Sci Adv. 2024 Oct 30;10(44):eado5924. doi: 10.1126/sciadv.ado5924 (PMC11524186; doi:10.1126/sciadv.ado5924)
Supplement: Supplementary file 1 — Supplementary Text Tables S1 to S8 Figs. S1 to S7 References [file sciadv.ado5924_sm.pdf]

Science Advances
AAAS

Supplementary Materials for

Calcite precipitation: The forgotten piece of lakes' carbon cycle

Gaël Many *et al.*

Corresponding author: Marie-Elodie Perga, marie-elodie.perga@unil.ch

Sci. Adv. **10**, eado5924 (2024)
DOI: 10.1126/sciadv.ado5924

This PDF file includes:

Supplementary Text
Tables S1 to S8
Figs. S1 to S7
References

1 Model settings

1.1 Model description

1.1.1 SIMSTRAT physical model

We used SIMSTRAT v3.0 (*28*), a one-dimensional physical lake model well suited for the simulation of stratification and mixing in deep stratified lakes (*41*). The model has been successfully applied to lakes under different conditions and over various periods (*42-45*). It combines a buoyancy-extended k–ε model with an internal seiche model to estimate the vertical diffusivity as a function of time and depth. The physical parameters used in the current version of the model are within the expected range and have been manually calibrated to match the temperature data collected at the Lake Geneva monitoring station between 1981 and 2021 (referred thereafter to as SHL2, see 2.2). Values used in this work are summarized in Table S1.

Model parameters	Value	Definition
**lat**	46.5	Latitude of the study area (°N)
**p_air**	969	Air pressure (mbar)
**a_seiche**	0.01	Fraction of wind energy which goes into seiche energy
**a_seiche_w**	0.003	Fraction of wind energy which goes into seiche energy in winter
**strat_sumr**	0.0001	If maximum Brunt-Väisälä frequency is below this threshold, a_seiche_w is used instead of a_seiche
**q_nn**	1	Fit parameter for distribution of seiche energy
**f_wind**	1	Ratio of forcing wind to wind speed at 10 m above lake level
**c10**	1	Wind drag coefficient
**cd**	0.002	Bottom drag coefficient
**hgeo**	0.12	Geothermal heat flux ($W.m^{-2}$)
**p_sw_water**	0.85	Fit parameter for absorption of short-wave radiation from sky
**p_lw**	0.97	Fit parameter for absorption of IR radiation from sky
**p_windf**	0.8	Fit parameter for convective and latent heat fluxes
**beta_sol**	0.35	Fraction of short-wave radiation directly absorbed as heat by water
**wat_albedo**	0.09	User defined water albedo
**p_albedo**	1	Fit parameter for albedo of ice, snow-ice and snow
**freez_temp**	0.01	Freezing temperature of water (°C)
**snow_temp**	2	Temperature of snow (°C)

Table S1. Physical parameters used in the version of SIMSTRAT in this work.

1.1.2 AED2 biogeochemical model

We used the Aquatic Ecodynamics model library (AED2, developed and maintained by the University of Western Australia, https://aed.see.uwa.edu.au/research/models/aed/, last visit on February 21, 2022, see details in (*29, 46*)), to reproduce the dynamics of the pelagic ecosystem in the water column with a daily time step over 1981-2021. The AED2 model is a multi-module biogeochemical model that was coupled to the physical model SIMSTRAT (https://github.com/Eawag-AppliedSystemAnalysis/Simstrat, last visit on May 25, 2023). The AED2 modules represent most aquatic biogeochemical processes, including nutrient cycling,

oxygen dynamics, sediment biogeochemistry, and the phyto- and zooplankton dynamics. All these modules were combined to reach the best representation of the C cycling in Lake Geneva, which is the main concern of this work.

For the purpose of this work, we used the following modules:

Oxygen module: Dissolved oxygen (DO) dynamics encompasses atmospheric exchanges and sediment oxygen demand (SOD). It also encompasses gross primary production (GPP) of O_2 and autotrophic respiration trough the *aed2_phytoplankton_module* (see below). The degradation of organic matter (OM) in the water column (i.e. from dissolved organic matter to dissolved inorganic matter), based on OM mineralization (i.e., heterotrophic respiration) is also linked to the DO dynamics in the *aed2_organic_matter* module. Both autotrophic and heterotrophic respirations are combined into total respiration (R in Fig. 2 of the main text) The SOD was set to 15 mmol O_2 m^{-2} d^{-1} and varies as a function of overlying water temperature and bottom water DO concentration (see online description for details, https://aquaticecodynamics.github.io/aed-science/DO_1.html, last visit on May 10, 2023). This value was set to fit to the bottom oxygen concentrations observed between 1981 and 2021 in the observations. This value is in line with previous works in Lake Geneva (10 mmol O_2 m^{-2} day^{-1} (*47*)) and Lake Lucerne (9 mmol O_2 m^{-2} day^{-1} (*48*)). The SOD was used to estimate the degradation of OM in the sediment and the related release of DIC using the oxygen-to-carbon molar ratio of 138:106 (*49*). Atmospheric exchanges were calculated from the gradient of DO with the atmosphere and gas transfer velocity computed according to Perolo et al. (2021) (*50*).

Nutrients (N/P/Si): The nitrogen, phosphorus, and silica cycles include the processes of biological uptake by phytoplankton specific groups into the internal nutrients pool (Si only by “diatoms” group; see below). Changes in nutrient pools also include dissolved sediment fluxes and phytoplankton mortality. The initial concentration of nutrients, sediment fluxes, and related parameters are synthesized in Table S2 and have been set to fit the observations between 1981 and 2021. Note that to simplify, we neglected the role of the organic matter mineralization in the hypolimnion (i.e., the aed2_organic_matter module, see below) and integrated this process inside each nutrient module through the release of N, P, and Si at the sediment-water interface.

**Model parameters**	**Value**	**Definition**
Oxygen module		
**oxy_initial**	NA	O_2 initial concentrations: profile from SHL2 (mmol O_2 m^{-3})
**Fsed_oxy**	-15	O_2 sediment flux (mmol m^{-2} day^{-1})
**theta_sed_oxy**	1.08	Arrhenius temperature multiplier for sediment O_2 flux
Nitrogen module		
**nit_initial**	38.5	NO_3 initial concentration (mmol N m^{-3})
**Fsed_nit**	2	NO_3 sediment flux (mmol N m^{-2} day^{-1})
**theta_sed_nit**	1.08	Arrhenius temperature multiplier for sediment NO_3 flux
Phosphorus module		
**frp_initial**	2.8	PO_4 initial concentration (mmol P m^{-3})
**Fsed_frp**	1.2	PO_4 sediment flux (mmol P m^{-2} day^{-1})
**theta_sed_frp**	1.08	Arrhenius temperature multiplier for sediment PO_4 flux
Silica module		
**rsi_initial**	35	Si initial concentration (mmol Si m^{-3})
**Fsed_rsi**	25	Si sediment flux (mmol Si m^{-2} day^{-1})
**theta_sed_rsi**	1.08	Arrhenius temperature multiplier for sediment Si flux

Table S2. Calibrated parameters used in the version of AED2 in this work.

Organic matter module: Inorganic and organic, dissolved and particulate forms of C, N and P are modelled in AED2 along the general degradation pathway from POM to DOM to dissolved inorganic matter (DIM). In this work, we chose to neglect the degradation of N and P, assuming that all dissolved inorganic nitrogen and phosphorus fluxes occur at the water-sediment interface (see above). However, we paid particular attention to C degradation (i.e., from particulate organic carbon (POC) to dissolved organic carbon (DOC) and from DOC to dissolved inorganic carbon (DIC)) to model the degradation of OC in the hypolimnion, a factor regulating DIC production and O_2 consumption at depth during the stratified period (*48, 51*). Parameters encompass the properties of POC (size, density and settling velocity), rates of hydrolyse/breakdown of POC to DOC, and mineralization of DOC to DIC. The parameters used are in line with the work of Bruce et al. (2006) (*52*), and are detailed in Table S3.

Model parameters	Value	Definition
POM to DOM		
**poc_initial**	8.3	POC initial concentration (mmol C m^{-3})
**Rpoc_hydrol**	0.01	hydrolysis/breakdown rate of POC to DOC (mmol C m^{-3} day^{-1})
**theta_hydrol**	1	Arrhenius temperature multiplier for POC breakdown
**w_pom**	-0.5	Settling velocity of POC (m day^{-1})
**d_pom**	0.000015	Diameter of POC (m)
**rho_pom**	1080	Density of POC (kg m^{-3})
DOM to DIM		
**doc_initial**	50	DOC initial concentration (mmol C m^{-3})
**Rdom_minerl**	0.001	Mineralization rate of DOC to DIC (mmol C m^{-3} day^{-1})
**theta_minerl**	1	Arrhenius temperature multiplier for DOC mineralization

Table S3. Parameters of the aed2_organic_matter module used in in this work.

Phyto- and Zooplankton modules: The algal biomass is simulated through several plankton functional types that are typically defined based on specific groups. Here, we chose to model the two mains groups of phytoplankton that are found in Lake Geneva, namely the “diatoms” and the “green” groups. The latter include the *Chlorophyceae* and *Zygnematophyceae* class. Each phytoplankton groups are simulated with their own growth, respiration, death and sedimentation processes (see Table S4). Distinction between groups is made by adoption of groups’ specific parameters for environmental dependencies, such as, growth temperature limitation, and nutrients fixation (C, Si, N, and P) (*53*). Zooplankton biomass is simulated through a unique group and the processes of food assimilation and losses from respiration, excretion, egestion, predation and mortality.

Model parameters	Value		Definition
General parameters			
**p_name**	***Diatoms***	***Green***	Groups of phytoplankton modelled in aed_phytoplankton
**p_initial**	0.1	0.1	Initial concentrations (mmol C m^{-3})
**p0**	0.03	0.03	Minimal concentrations (mmol C m^{-3})
**w_p**	-0.1	-0.1	Settling velocities (m d^{-1})
**Xcc**	40	40	Carbon to chlorophyll ratio (mg C.mg $chla^{-1}$)
Growth parameters			
**R_growth**	2.0	1.1	Phyto max growth rate @20°C (d^{-1})
**fT_Method**	1	1	Temperature limitation function of growth (1=CAEDYM style)
**theta_growth**	1	1	Arrenhius temperature scaling for growth function

**T_std**	7	18	Standard temperature (°C)
**T_opt**	13	24	Optimum temperature (°C)
**T_max**	30	30	Maximum temperature (°C)
Light parameters			
**lightModel**	0	0	Type of light response function (0 = no photoinhibition)
**I_K**	4	15	Half saturation constant for light limitation of growth (µE m^{-2} s^{-1})
**KePHY**	0.001	0.001	Specific attenuation coefficient
Respiration parameters			
**f_pr**	0.001	0.001	Fraction of primary production lost to exudation
**R_resp**	0.04	0.11	Phytoplankton respiration/metabolic loss rate @20°C
**theta_resp**	1	1	Arrhenius temperature scaling factor for respiration
**k_fres**	0.70	0.70	Fraction of metabolic loss that is true respiration
**k_fdom**	0.15	0.15	Fraction of metabolic loss that is DOM
Nitrogen parameters			
**N_0**	0.25	0.25	Nitrogen concentration below which uptake is 0 (mmol N m^{-3})
**K_N**	3.5	3.5	Half-saturation concentration of nitrogen (mmol N m^{-3})
**X_nmin**	0.01	0.01	Minimum internal nitrogen concentration (mmol N. mmol C^{-1})
**X_nmax**	0.2	0.2	Maximum internal nitrogen concentration (mmol N. mmol C^{-1})
**R_nuptake**	0.08	0.08	Maximum nitrogen uptake rate(mmol N m^{-3} d^{-1})
**R_nfix**	0.1	0.1	Nitrogen fixation rate
Phosphorous parameters			
**P_0**	0.0645	0.0645	Phosphorus concentration below which uptake is 0 (mmol P m^{-3})
**K_P**	0.15	0.15	Half-saturation concentration of phosphorus (mmol P m^{-3})
**X_pmin**	0.0001	0.0001	Minimum internal phosphorus concentration (mmol P. mmol C^{-1})
**X_pmax**	0.015	0.015	Maximum internal phosphorus concentration (mmol P. mmol C^{-1})
**R_puptake**	0.01	0.01	Maximum phosphorus uptake rate (mmol P m^{-3} d^{-1})
Silica parameters			
**Si_0**	0.1	NA	Silica concentration below which uptake is 0 (mmol Si m^{-3})
**K_Si**	2.5	NA	Half-saturation concentration of silica (mmol Si m^{-3})
**X_sicon**	0.4	NA	Constant internal silica concentration (mmol Si. mmol C^{-1})

Table S4. AED2 phytoplankton groups parameters (aed2_phytoplankton.nml). General, growth, light, respiration, nitrogen, phosphorous and silica parameters are specified for the diatoms and green phytoplankton groups.

1.1.3 Description of the carbon and calcite modules

Carbon module: A specific description of the *aed2_carbon* module is presented thereafter. The core variable in the module is the DIC. DIC dynamics is impacted inside the module by exchanges at the air-water (i.e., CO_2 flux) and sediment-water (i.e., SOD- and calcite dissolution-induced DIC fluxes) interfaces, and by calcite precipitation (CP; see below). DIC is also impacted through other modules for primary production (*aed2_phytoplankton, aed2_zooplankton*) and by the hydrolysis and bacterial mineralization of the OM (i.e., the *aed2_organic_matter* module, see above). In this work, we neglected the dynamics of methane (CH_4) fluxes at the sediment and air interfaces and CH4 oxidation, as the deep production of methane and surface methane concentrations are negligible in Lake Geneva.

Following the calculation of the DIC, balanced by the different organic and inorganic processes, the base of the module consists in estimating the remaining parameters of the carbonate system, i.e., the total alkalinity (TAC), the pH and the CO_2 concentration. In this work, we chose to estimate TAC from calcium concentration (expressed in mmol m^{-3}) as these two variables are strongly linearly related in Lake Geneva. Inversely, TAC is not a linear function of DIC in Lake

Geneva due to the uptake of CO_2 by primary producers. A specific work has been done to express TAC independently of the DIC. The linear relationship used between TAC and calcium, from in-situ observations is shown in Figure S1. The relation obtained and set in the model is in line with the previous work of Escoffier et al. (2023) (*26*). The pH and related CO_2 concentration are then estimated using the CO2SYS (*54*) using temperature, salinity, DIC and TAC, and the specific constants from Millero (1979) (*55*) for freshwaters. Note that for air-water exchanges of CO_2, the same gas piston model as the one in the oxygen module was used (see above).

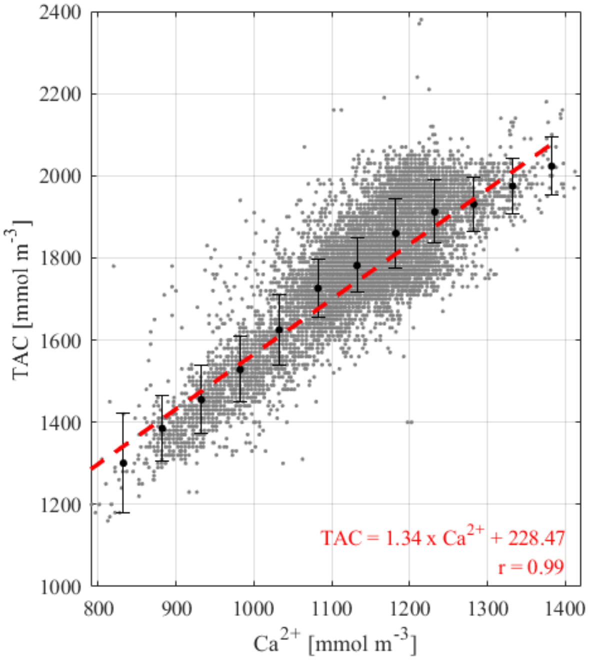

Fig. S1. Relation used to express TAC as a function of Ca^{2+} concentration in the "aed2_carbon" module. Data used have been monitored at the SHL2 measuring station between 1981 and 2021. Here, TAC is expressed in mmol m^{-3} to fit with the model parametrization.

Calcite precipitation – In Lake Geneva, the dynamics of TAC and related DIC, are affected by calcite precipitation (CP) (*26, 56*). We added a module for CP within the *aed2_carbon* module. This module consists of calculating CP fluxes with respect to the saturation index of calcite, Ω. First, the model defines CP reaction in terms of components. An aqueous species $CaCO_3$ is created as a product of reaction between components Ca^{2+} and CO_3^{2-}:

$Ca^{2+} + CO_3^{2-} \leftrightarrow CaCO_{3(s)}$

Ca^{2+} is defined as an initial condition, i.e. a state variable, expressed in mmol m^{-3} from the SHL2 data in January 1981. CO_3^{2-} is estimated from the speciation of the simulated DIC concentrations using the pH at the corresponding time step. Then, the calcite saturation index Ω is calculated from the Ion Activity Product (IAP) and the temperature-corrected solubility constant Ks according to the following formula: $\Omega = IAP/Ks$. IAP is computed from the product of Ca^{2+} and CO_3^{2-} activities, (i.e., $\{Ca^{2+}\}$ and $\{CO_3^{2-}\}$) using the activity constant derived using the Güntelberg method. Ks constant is adjusted to modeled temperatures (T, in K) according to Plummer and Busenberg (1982) (*57*):

$log(Ks) = -171.9065 - 0.077993 * T + 2839.319/T + 71.595*log(T)$

At last, Ω is used to define conditions where calcite precipitates. Calcite precipitates for Ω>1.5, a threshold value previously shown as a minimum calcite supersaturation level allowing for potential precipitation in Lake Geneva and other nearby peri-alpine lakes (22, *26, 57-58*). Additionally, a specific filter of T = 15°C is used to onset the CP, as this reaction can only occur above this temperature threshold, in the physico-chemical conditions of Lake Geneva (*59*) The carbon flux from which $CaCO_3$ precipitates was set to 2.5 mmol C m^{-3} d^{-1}, based on in-situ observations for lake Geneva (18). This estimate is also in line with the previous works (*4, 15*). The settling velocity of the precipitated calcite was set to 10 mm s^{-1} (*60*).

Calcite dissolution – We computed the dissolution of calcite through the release of Ca^{2+} estimated at the sediment-water interface. A flux of 7 mmol m^{-2} d^{-1} of Ca^{2+} was set to fit to the observed Ca^{2+} bottom concentrations at SHL2 from 1981 to 2021. The C-derived flux estimated from calcite dissolution was then added to the release flux of DIC. The latter thus combines the release of DIC from the OM mineralization (estimated using the SOD, see 1.1.2), and the release of DIC from the dissolution of calcite using the Ca^{2+} sediment flux (CD in Fig. 2 of the main text).

1.2 Data source for model setting and validation

1.2.1 Initial conditions, meteorological forcings and inflows

The model's initial conditions are set following the corresponding observations at the SHL2 monitoring station. We defined the values of physical and biogeochemical parameters from the profile carried out at the SHL2 station in January 1981. Meteorological conditions from 1981 to 2021 were downloaded from the MeteoSwiss IDAWEB website (https://gate.meteoswiss.ch/idaweb/login.do) and are used to force the model. The wind speed and direction, the air temperature, the solar radiation, the air vapor content, and the percentage of cloud at an hourly resolution are measured at the Geneva-Cointrin station (code station GVE; 6°08'E; 46°15'N).

The Rhône River Inflows was considered as the sole inflow, i.e. we neglected the inflows from the Dranse, Aubonne, Venoge, and several other small rivers. The Rhône River is responsible for 70-75% of the total inflow, with a mean discharge of 187 m^3 s^{-1}. The Rhône discharge, physical (temperature), and biogeochemical conditions (expressed in mmol m^{-3} s^{-1} for NO_3, PO_4, DO, DIC, POC, Ca^{2+}) were downloaded from the FOEN website (*61*). The parameters are monitored bi-monthly at Porte du Scex station. To fit with the model's resolution, we interpolated the different parameters on a daily resolution. Note that for missing values (mainly between 2018 and 2021, as the data were not available), we used the general relationship between the variable of interest and the river discharge. The detail of the Rhone River conditions for the period simulated is presented in Figure S2.

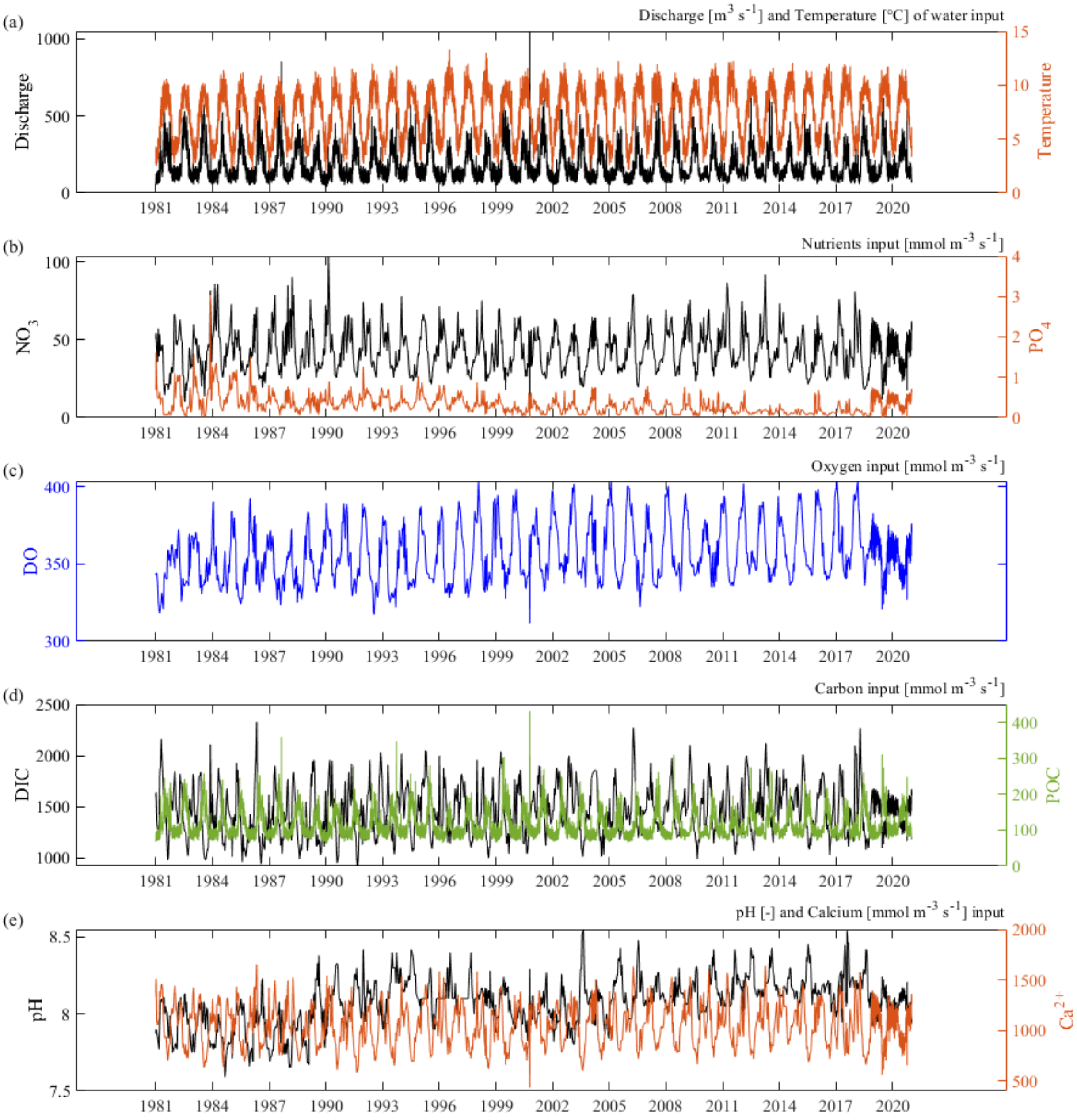

Fig. S2. Detail of the Rhone River inputs between 1981 and 2021.

1.2.2 In situ observations of Lake Geneva

Since 1957, the physical and biogeochemical conditions of Lake Geneva have been measured on a regular basis by the Commission International pour la Protection des Eaux du Léman (CIPEL). In this work, several variables were used to parametrize and validate the simulations (see Table S5). These variables were monitored at the deepest point of the lake (SHL2) throughout the entire water column for 8–12 times per year between 1957 and 1970 and at least once per month thereafter to present (© OLA-IS, AnaEE-France, INRAE of Thonon-les-Bains, CIPEL (*30*)). The CO2SYS is used to estimate the DIC concentration (in mmol C m^{-3}) and corresponding pCO_2

(expressed in ppm) from the monitored temperature, TAC, pH, and the specific constants for freshwaters.

Variables	Units	Monitored or Estimated	Period	Depth
**Temperature**	°C	Monitored	1981-2021	0-309m
**Dissolved Oxygen**	mg O_2 L^{-1}	Monitored	1981-2021	0-309m
**Nitrate**	mg N L^{-1}	Monitored	1981-2021	0-309m
**Phosphate**	mg P L^{-1}	Monitored	1981-2021	0-309m
**Silica**	mg Si L^{-1}	Monitored	1981-2021	0-309m
**Calcium**	mg Ca^{2+} L^{-1}	Monitored	1981-2021	0-309m
**TAC**	mmol L^{-1}	Monitored	1981-2021	0-309m
**pH**	-	Monitored	1981-2021	0-309m
**DIC**	mmol C m^{-3}	Estimated	1981-2021	0-309m
**pCO_2**	ppm	Estimated	1981-2021	0-309m
**POC**	mg C L^{-1}	Monitored	1986-2021	0-309m

Table S5. Variables from SHL2 monitoring station used to parametrize and validate the model.

2 Model validation

2.1 Overview

Here, we show that the model parametrized and used in this work is faithful to the reality of the observations made at SHL2 between 1981 and 2021. The variables used for the validation of the model are gathered in a Taylor diagram in Figure S3, and the full dataset is presented in Figure S4. Overall, the model faithfully reproduces the dynamics of the lake in terms of physical structure (temperature) and nutrient dynamics (N, P, Si). Moreover, the dynamic of the carbonate system (DIC, pH, pCO_2, TAC) is well represented by the model with correlation coefficients vs. observations $r \geq 0.6$. The calcite module implemented in the carbon module allows a good description, as shown by the calcium dynamics in the lake with r = 0.7. All the validation parameters used (correlation coefficient, standard deviation, centered root mean square error) are summarized in Table S6.

Variable	Units	Mean obs.	Std obs.	Mean mod.	Std mod.	r vs. obs.	RMSE vs. obs.
**Temperature**	°C	6.29	1.47	6.18	1.52	0.96	0.27
**Dissolved Oxygen**	mg O_2 L^{-1}	8.10	1.79	7.24	1.70	0.83	1.11
**Nitrate**	mg N L^{-1}	0.56	0.05	0.51	0.08	0.16	0.08
**Phosphate**	mg P L^{-1}	0.04	0.02	0.05	0.02	0.88	0.01
**Silica**	mg Si L^{-1}	2.35	0.94	1.88	0.82	0.79	0.66
**POC**	mg C L^{-1}	0.08	0.06	0.03	0.04	0.38	0.06
**DIC**	mmol C m^{-3}	1902.8	104.4	1939.1	117.8	0.86	56.9
**TAC**	mmol L^{-1}	1.82	0.08	1.81	0.07	0.86	0.03
**pH**	-	7.82	0.12	7.70	0.17	0.63	0.17
**pCO_2**	ppm	1513.6	359.3	2075.5	714.4	0.58	723.4
**Calcium**	mmol Ca m^{-3}	1159.9	40.2	1185.2	52.8	0.70	44.9

Table S6. Parameters used for the validation of the model.

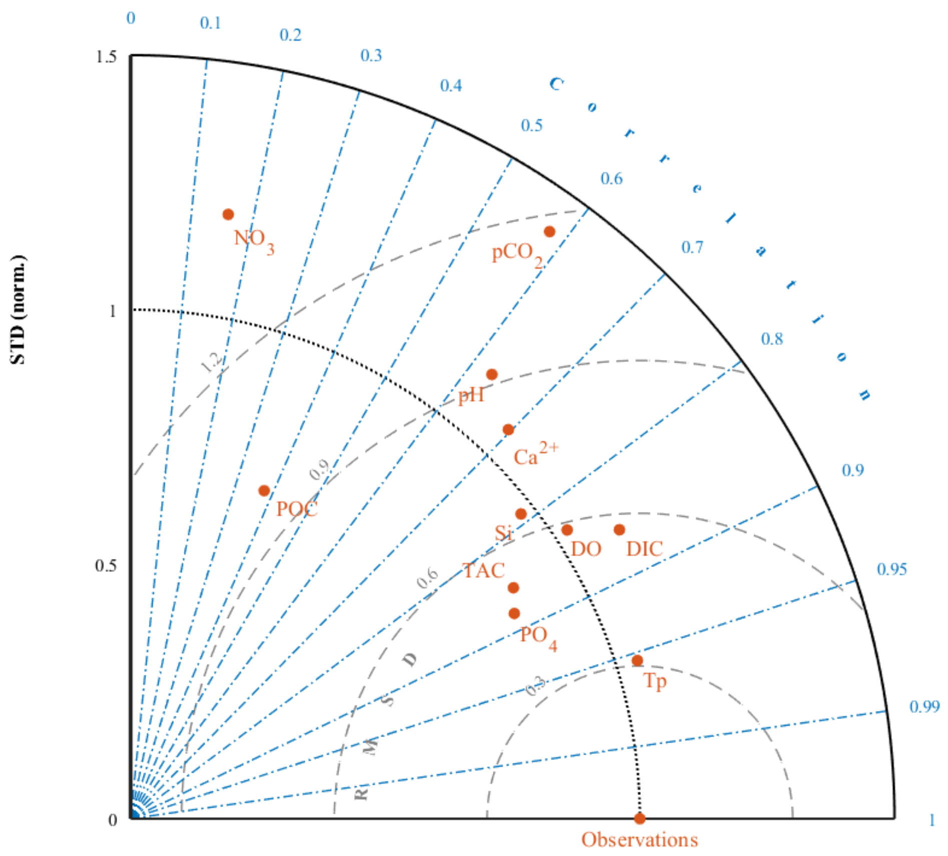

Fig. S3. Taylor diagram of the different parameters used to validate the model. Note that we normalized the results to compare all the variables in a unique Taylor diagram.

To go further, Figure S4 shows the full dataset used to validate the model in terms of temperature, nutrients, oxygen, C system (POC, DIC, TAC, pH, and pCO2), and calcium (daily profiles between 0 and 309m between 1981 and 2021). The model reproduces well the periods of high surface temperature during the summer (Fig. S4a), leading to the increased stratification and nutrient uptake by the phytoplankton community (Fig. S4c-e). This leads to an increase in POC (Fig. S4f) and surface oxygen (Fig. S4b), in line with a decrease in surface DIC (Fig. S4g). At the same time, the decrease in TAC is well reproduced during the stratified periods and correlates to the increase in pH and decrease in pCO_2 (Fig. S4h-j). Finally, the simulated CP, validated by the summer decreases in surface calcium, is in agreement with the observations (Fig. S4k).
Regarding winter dynamics, the model reproduces the complete mixing periods observed in 1985, 1986, 1999, 2000, 2005, 2006, and 2012. The periods of weak winter mixing are also reproduced, mainly over the periods 1982-1984, 1990-1998, and 2013-2021, resulting in a period of hypoxia at the lake bottom (Fig. S4b, DO < 4 mg L^{-1}), and an increase of the bottom DIC and pCO_2 (Fig. S4g and S4j). These observations are consistent with the work of Schwefel et al. (*47, 62*).

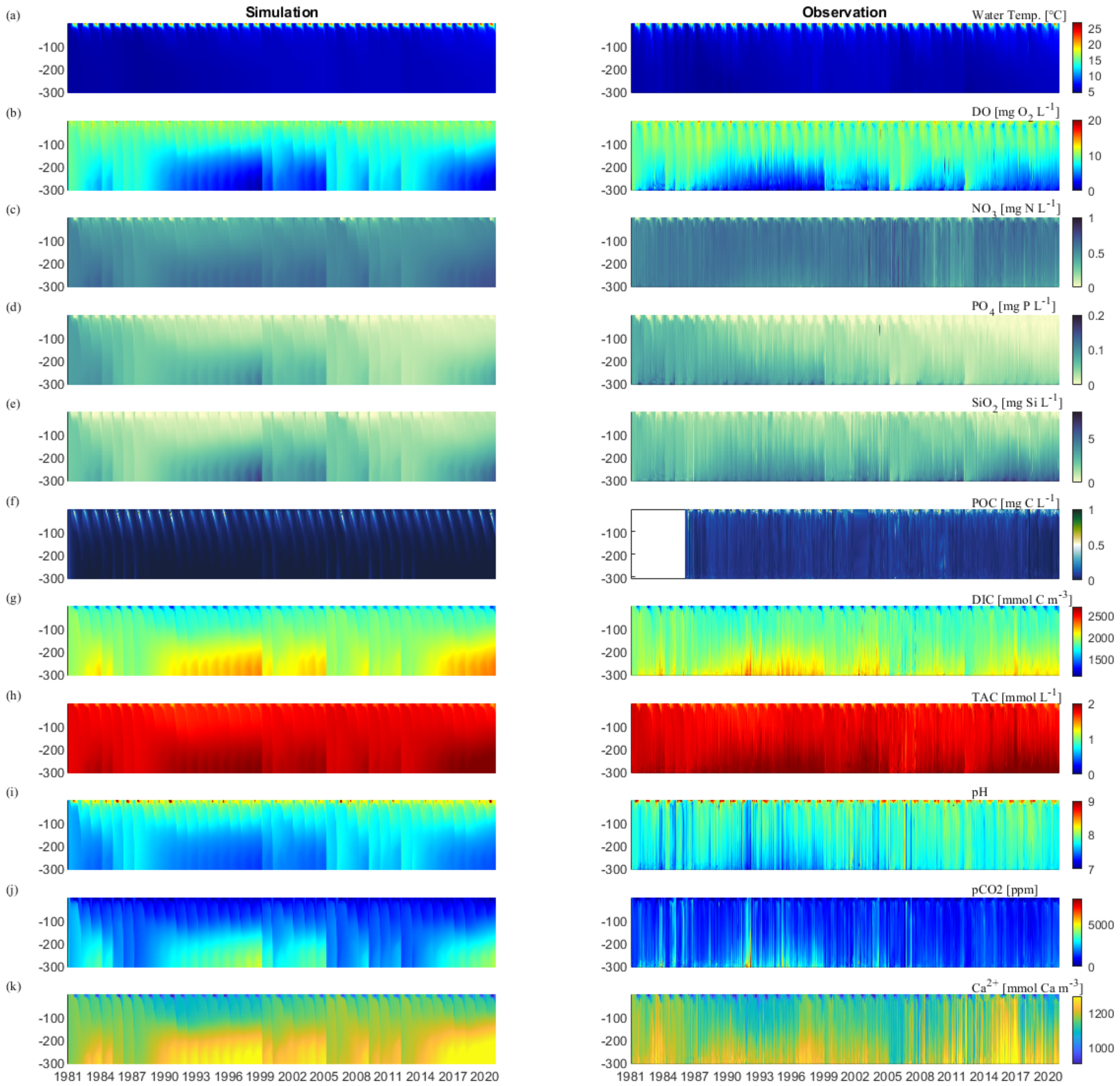

Fig. S4. Modeled (left) and measured (right) profiles between 1981 and 2021 of (a) temperature (°C), (b) DO (mg O_2 L^{-1}), (c) NO3 (mg N L^{-1}), (d) PO4 (mg P L^{-1}), (e) SiO_2 (mg Si L^{-1}), (f) POC (mg C L^{-1}), (g) DIC (mmol C m^{-3}), (h) TAC (mmol L^{-1}), (i) pH, (j) pCO_2 (ppm), (k) Ca^{2+} (mmol Ca m^{-3}).

2.2 *Physical validation using the modeled temperature.*

Comparisons of simulated and measured temperatures at SHL2 in Lake Geneva for 1981-2021 are presented in Figure S5 (SIMSTRAT v3.0 model). The average of the modeled temperature, 6.18°C, is very close to the average of measured temperatures, 6.29°C. The correlation between the two datasets (r=0.96) shows that the model reproduces well the seasonal variability of the lake temperature, also visible on the surface time series (0-10m depth, Fig. S5a). The RMSE for surface waters of 0.97°C (0-10m) and for bottom waters of 0.20°C (300-310m, Fig. S5b), are satisfying and in agreement with previous works (32). Main differences are found close to the thermocline during the stratified period (Fig. S5c). Baroclinic motions, such as by internal seiche or gyres, deflect the thermal structure locally but cannot be accounted for in a 1D vertical model (*62*).

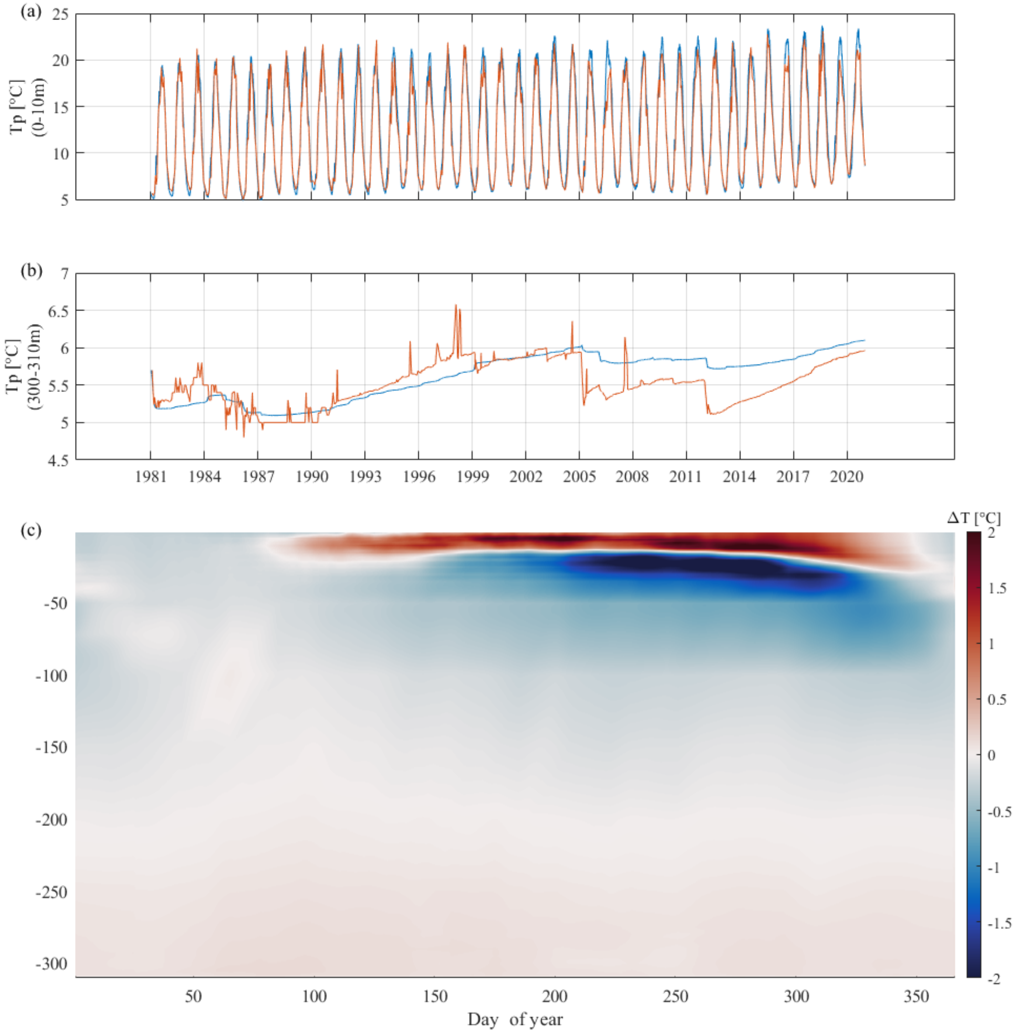

Fig. S5. Modeled (blue) and measured (orange) temperatures 1981–2021 between (a) 0-10m depth and (b) 300-310m depth. c) Deviation of the annual modeled temperatures from the measured values (1981–2021).

2.3 Validation for biogeochemical parameters at the lake surface

We detail here the validation of the surface conditions as most of the processes regulating carbon uptake occur at the surface (i.e. primary production and CP). Comparisons of the climatology of simulated and measured surface conditions for the period 1981-2021 are presented in Figure S6 (averaged on 0-10m depth). The beginning of spring leads to an increase in surface temperatures and stratification. In parallel, the uptake of DIC by primary production and TAC within CP occurs. Phytoplankton activity leads to a decrease in available nutrient concentrations, which is related to an increase in surface DO and POC. CP also leads to a decrease of Ca^{2+} concentrations. As the lake stratifies, CO_2 equilibration with the atmosphere and the enhanced primary production deplete CO_2 on the surface and lead to an increase in pH. In late autumn, the onset of the winter period leads to a drop in surface temperatures and triggers convective mixing. The latter remobilizes deep nutrients and C, replenishing surface DIC, TAC, CO_2, and calcium. All the simulated variables show a good correlation with the observations made at SHL2 (r>0.6). Furthermore, the errors are small (see σ in Fig. S6), showing that the simulated processes are in good agreement with the observations.

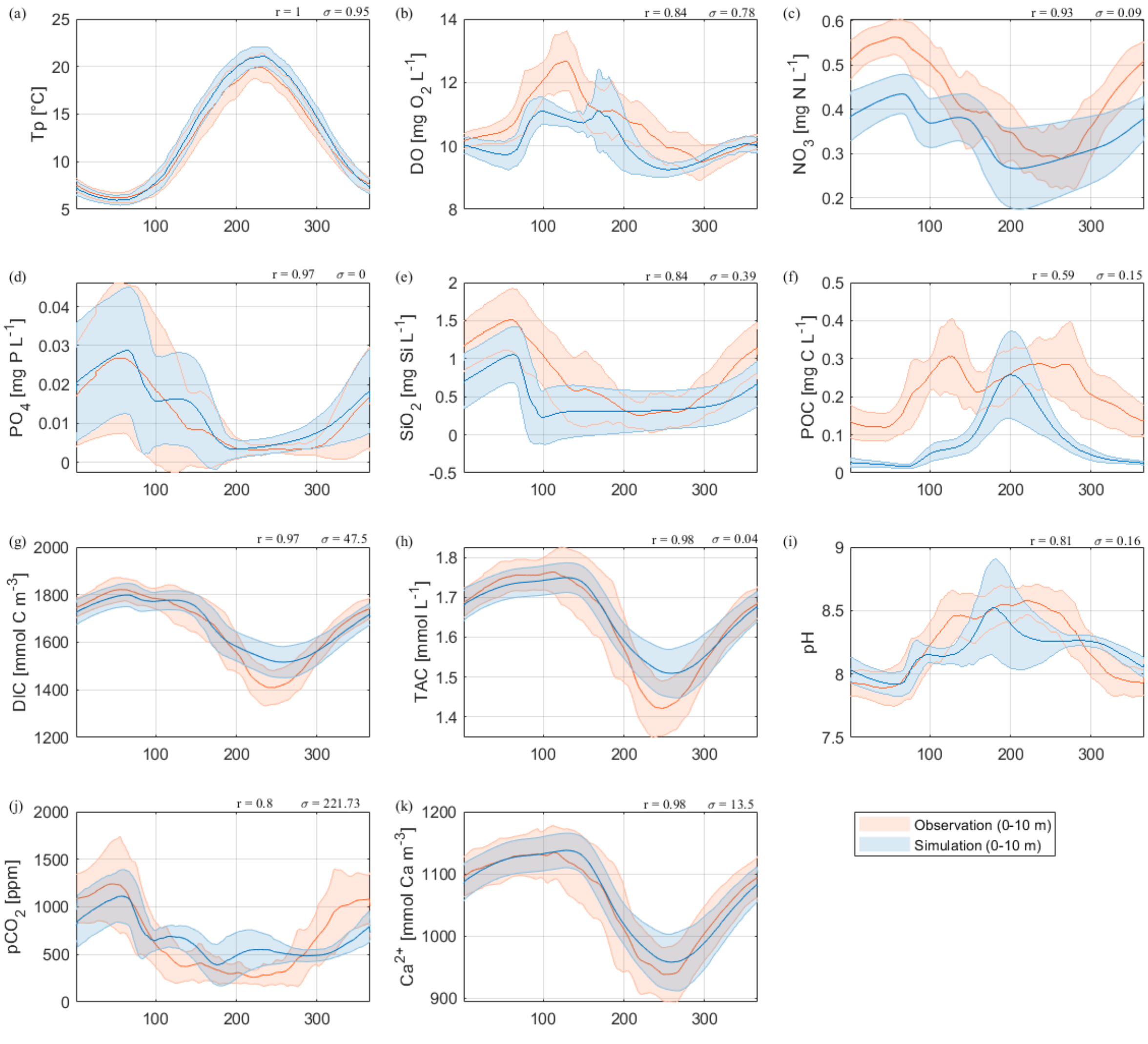

Fig. S6. Climatology (expressed as day of year_DOY) of surface modeled (blue) and measured (orange) variables on 1981–2021. The correlation coefficient r and the RMSE σ are specified on each plot.

2.4 Validation of the calcite module

As explained in 1.1.3, a module coding for the calcite dynamics was implemented in the carbon module. To validate our simulations, we used the calcium data measured at SHL2 between 1981 and 2021 (expressed in mmol Ca m^{-3} (*26*). Figure S7 shows the comparison between simulated and measured calcium concentrations at the lake surface (Fig. S7a), and bottom (Fig. S7b). Note that in this work, we neglected the dissolution of calcite in the water column and assumed that calcite dissolution only occurs in the sediment. The RMSE for surface waters of 48 mmol Ca m^{-3} (0-10m) and bottom waters of 65.9 mmol Ca m^{-3} (300-310m) are satisfying and suggest that the model reproduces the changes in calcium conditions induced by CP during the stratified period and calcite dissolution from the sediment all year long.

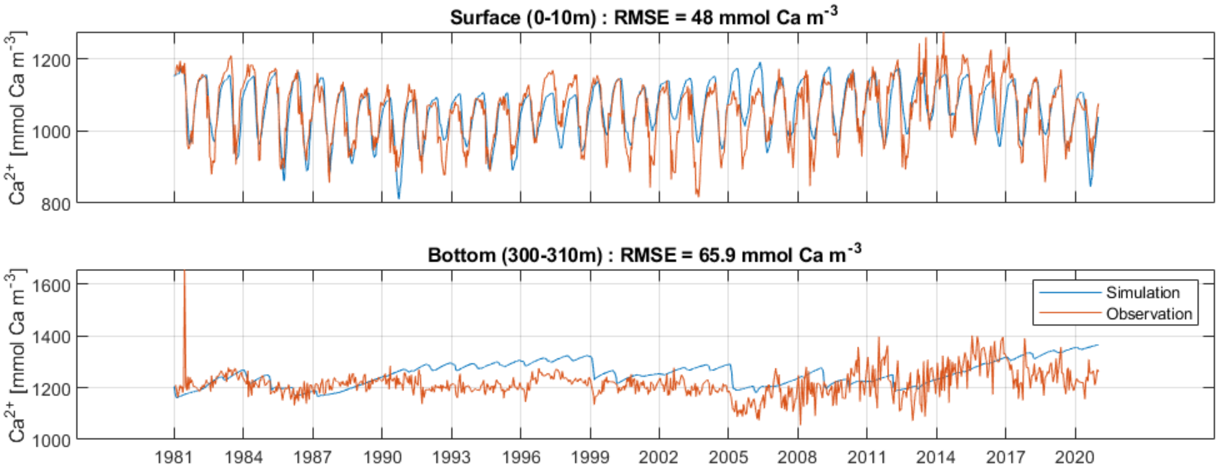

Figure S7. Time-series of simulated (blue) and observed (orange) calcium concentrations (expressed in mmol Ca m^{-3}) in Lake Geneva for (a) surface waters (0-10 m depth) and (b) bottom waters (300-310m). The RMSE is specified.

2.5 Test cases

Specific experiments were carried out to assess how much the model calibrated for Lake Geneva can be expanded to other hardwater lakes. The initial alkaline conditions were changed over a range of ca. 0.35 to 3.5 mol m^{-3} (see Table S7). This allowed us to estimate the role that the alkalinity level, mediated by CP, plays on the surface CO_2 supersaturation (Ω_{CO2}). Conversely, a reduction in alkalinity was tested to determine the threshold for which CO_2 oversaturation is reached (Ω_{CO2} = 1). These experiments were carried out over four arbitrary periods of 5 years (1981-1985; 1991-1995; 2001-2005; and 2011-2015) to avoid long-term re-equilibrium due to organic matter processes such as water column and sediment respiration. Note that for these experiments using the model without CP, the sediment release of Ca^{2+} and DIC from calcite dissolution was switched off (i.e., the 7 mmol C m^{-2} d^{-1}; see 1.1.3). Only the sediment organic

respiration was considered (based on the SOD, 15 mmol O_2 m^{-2} d^{-1}; see 1.1.2). Besides, the Rhône River inputs in terms of Ca^{2+} and DIC were also adapted to fit the initial conditions of the lake.

Test case	Level of alkalinity (mol m^{-3})	Mean ini. DIC (mol m^{-3})	Mean ini. Ca^{2+} (mol m^{-3})
**1**	0.35	0.380	0.230
**2**	0.87	0.955	0.575
**3***	1.75	1.920	1.150
**4**	3.50	3.840	2.300

Table S7. Parameters used for the test cases on the level of alkalinity. Initial conditions in terms of DIC and Ca^{2+} concentrations are specified. * Test case number 3 is the reference simulation.

We then compared the simulations to 18 other lake basins in Switzerland and US, whose geochemical conditions are shown in Table S8.

Lake name	Country	Alk. (mol m^{-3})	Ω_{CO2}
**Baldegg**	CH	3.31	3.79
**Brienz**	CH	1.44	1.57
**Hallwil**	CH	2.82	3.39
**Lucerne (Alpnachersee)**	CH	2.88	3.53
**Lucerne (Kreuztrichter)**	CH	1.86	1.84
**Lucerne (Urnersee)**	CH	1.79	1.54
**Lugano (North)**	CH	2.06	1.54
**Lugano (South Figino)**	CH	1.93	2.44
**Neuchatel**	CH	2.55	3.52
**Pfaeffikon**	CH	3.60	3.79
**Sempachersee**	CH	2.35	2.24
**Walenstadt**	CH	2.15	2.02
**Zug**	CH	2.55	2.06
**Zurich**	CH	2.49	2.41
**Erie**	US	1.86	2.30
**Huron**	US	1.68	1.12
**Michigan**	US	2.18	2.60
**Ontario**	US	1.79	1.49

Table S8. Geochemical conditions of the 18 lake basins used for comparison with the simulations.

REFERENCES AND NOTES

1. J. J. Cole, Y. T. Prairie, N. F. Caraco, W. H. McDowell, L. J. Tranvik, R. G. Striegl, C. M. Duarte, P. Kortelainen, J. A. Downing, J. J. Middelburg, J. Melack, Plumbing the global carbon cycle: Integrating inland waters into the terrestrial carbon budget. *Ecosystems* **10**, 172–185 (2007).

2. L. J. Tranvik, J. J. Cole, Y. T. Prairie, The study of carbon in inland waters—From isolated ecosystems to players in the global carbon cycle. *Limnol. Oceanogr. Lett.* **3**, 41–48 (2018).

3. C. Le Quéré, R. M. Andrew, P. Friedlingstein, S. Sitch, J. Hauck, J. Pongratz, P. A. Pickers, J. I. Korsbakken, G. P. Peters, J. G. Canadell, A. Arneth, V. K. Arora, L. Barbero, A. Bastos, L. Bopp, F. Chevallier, L. P. Chini, P. Ciais, S. C. Doney, T. Gkritzalis, D. S. Goll, I. Harris, V. Haverd, F. M. Hoffman, M. Hoppema, R. A. Houghton, G. Hurtt, T. Ilyina, A. K. Jain, T. Johannessen, C. D. Jones, E. Kato, R. F. Keeling, K. K. Goldewijk, P. Landschützer, N. Lefèvre, S. Lienert, Z. Liu, D. Lombardozzi, N. Metzl, D. R. Munro, J. E. M. S. Nabel, S. Nakaoka, C. Neill, A. Olsen, T. Ono, P. Patra, A. Peregon, W. Peters, P. Peylin, B. Pfeil, D. Pierrot, B. Poulter, G. Rehder, L. Resplandy, E. Robertson, M. Rocher, C. Rödenbeck, U. Schuster, J. Schwinger, R. Séférian, I. Skjelvan, T. Steinhoff, A. Sutton, P. P. Tans, H. Tian, B. Tilbrook, F. N. Tubiello, I. T. van der Laan-Luijkx, G. R. van der Werf, N. Viovy, A. P. Walker, A. J. Wiltshire, R. Wright, S. Zaehle, B. Zheng, Global carbon budget 2018. *Earth Syst. Sci. Data* **10**, 2141–2194 (2018).

4. M. E. Perga, S. C. Maberly, J. P. Jenny, B. Alric, C. Pignol, E. Naffrechoux, A century of human-driven changes in the carbon dioxide concentration of lakes. *Global Biogeochem. Cycles* **30**, 93–104 (2016).

5. G. A. Weyhenmeyer, S. Kosten, M. B. Wallin, L. J. Tranvik, E. Jeppesen, F. Roland, Significant fraction of CO_2 emissions from boreal lakes derived from hydrologic inorganic carbon inputs. *Nat. Geosci.* **8**, 933–936 (2015).

6. P. A. Del Giorgio, J. J. Cole, N. F. Caraco, R. H. Peters, Linking planktonic biomass and metabolism to net gas fluxes in northern temperate lakes. *Ecology* **80**, 1422–1431 (1999).

7. C. M. Duarte, Y. T. Prairie, Prevalence of heterotrophy and atmospheric CO_2 emissions from aquatic ecosystems. *Ecosystems* **8**, 862–870 (2005).

8. N. R. Urban, A. Desai, Are the Great Lakes a significant net source or sink of CO_2? *SIL Proc.* **30**, 1283–1288 (2009).

9. M. Rantakari, P. Kortelainen, Controls of organic and inorganic carbon in randomly selected Boreal lakes in varied catchments. *Biogeochemistry* **91**, 151–162 (2008).

10. E. G. Stets, R. G. Striegl, G. R. Aiken, D. O. Rosenberry, T. C. Winter, Hydrologic support of carbon dioxide flux revealed by whole-lake carbon budgets. *J. Geophys. Res. Biogeo.* **114**, G01008 (2009).

11. S. C. Maberly, P. A. Barker, A. W. Stott, M. M. De Ville, Catchment productivity controls CO_2 emissions from lakes. *Nat. Clim. Change* **3**, 391–394 (2013).

12. D. Langmuir, *Aqueous Environmental Geochemistry* (Prentice-Hall, Inc., 1997).

13. R. Marcé, B. Obrador, J.-A. Morguí, J. Lluís Riera, P. López, J. Armengol, Carbonate weathering as a driver of CO_2 supersaturation in lakes. *Nat. Geosci.* **8**, 107–111 (2015).

14. C. P. McDonald, E. G. Stets, R. G. Striegl, D. Butman, Inorganic carbon loading as a primary driver of dissolved carbon dioxide concentrations in the lakes and reservoirs of the contiguous United States. *Global Biogeochem. Cycles* **27**, 285–295 (2013).

15. H. Khan, R. Marcé, A. Laas, B. Obrador, The relevance of pelagic calcification in the global carbon budget of lakes and reservoirs. *Limnetica* **41**, 17–25 (2022).

16. A. E. Strong, B. J. Eadie, Satellite observations of calcium carbonate precipitations in the Great Lakes 1. *Limnol. Oceanogr.* **23**, 877–887 (1978).

17. N. Pasche, G. Alunga, K. Mills, F. Muvundja, D. B. Ryves, M. Schurter, B. Wehrli, M. Schmid, Abrupt onset of carbonate deposition in Lake Kivu during the 1960s: Response to recent environmental changes. *J. Paleolimnol.* **44**, 931–946 (2010).

18. N. Gu, W. Jiang, L. Wang, E. Zhang, S. Yang, S. Xiong, Rainfall thresholds for the precipitation of carbonate and evaporite minerals in modern lakes in northern China. *Geophys. Res. Lett.* **42**, 5895–5901 (2015).

19. B. W. Bird, M. B. Abbott, M. Vuille, D. T. Rodbell, N. D. Stansell, M. F. Rosenmeier, A 2,300-year-long annually resolved record of the South American summer monsoon from the Peruvian Andes. *Proc. Natl. Acad. Sci. U.S.A.* **108**, 8583–8588 (2011).

20. J. Küchler-Krischun, J. Kleiner, Heterogeneously nucleated calcite precipitation in Lake Constance. A short time resolution study. *Aquat. Sci.* **52**, 176–197 (1990).

21. R. O. Megard, Planktonic photosynthesis and the environment of calcium carbonate deposition in lakes. *Int. Ver. Theor. Angew. Limnol. Mitt.* **17**, 94 (1969).

22. M. Dittrich, M. Obst, Are picoplankton responsible for calcite precipitation in lakes? *AMBIO J. Hum. Environ.* **33**, 559–564 (2004).

23. T. A. McConnaughey, J. W. LaBaugh, D. O. Rosenberry, R. G. Striegl, M. M. Reddy, P. F. Schuster, V. Carter, Carbon budget for a groundwater-fed lake: Calcification supports summer photosynthesis. *Limnol. Oceanogr.* **39**, 1319–1332 (1994).

24. E. S. Homa, S. C. Chapra, Modeling the impacts of calcite precipitation on the epilimnion of an ultraoligotrophic, hard-water lake. *Ecol. Model.* **222**, 76–90 (2011).

25. P. Perolo, N. Escoffier, H. E. Chmiel, G. Many, D. Bouffard, M.-E. Perga, Alkalinity contributes at least a third of annual gross primary production in a deep stratified hardwater lake. *Limnol. Oceanogr. Lett.* **8**, 359–367 (2023).

26. N. Escoffier, P. Perolo, G. Many, N. T. Pasche, M.-E. Perga, Fine-scale dynamics of calcite precipitation in a large hardwater lake. *Sci. Total Environ.* **864**, 160699 (2023).

27. M. Ishikawa, W. Gonzalez, O. Golyjeswski, G. Sales, J. A. Rigotti, T. Bleninger, M. Mannich, A. Lorke, Effects of dimensionality on the performance of hydrodynamic models for stratified lakes and reservoirs. *Geosci. Model Dev.* **15**, 2197–2220 (2022).

28. G. H. Goudsmit, H. Burchard, F. Peeters, A. Wüest, Application of k-ϵ turbulence models to enclosed basins: The role of internal seiches. *J. Geophys. Res. Oceans* **107**, 23-1–23-13 (2002).

29. M. R. Hipsey, C. Boon, D. Paraska, L. C. Bruce, P. Huang, AquaticEcoDynamics/libaed2: v1.3.0-rc2. In Aquatic EcoDynamics (AED) Model Library & Science Manual (v1.3.0, p. 1-34). The University of Western Australia Technical Report (2019); https://doi.org/10.5281/zenodo.2538495.

30. F. Rimet, O. Anneville, D. Barbet, C. Chardon, L. Crépin, I. Domaizon, J.-M. Dorioz, L. Espinat, V. Frossard, J. Guillard, C. Goulon, V. Hamelet, J.-C. Hustache, S. Jacquet, L. Lainé, B. Montuelle, P. Perney, P. Quetin, S. Rasconi, A. Schellenberger, V. Tran-Khac, G. Monet, The Observatory on LAkes (OLA) database: Sixty years of environmental data accessible to the public: The Observatory on LAkes (OLA) database. *J. Limnol.* **79**, 164–178 (2020).

31. L. M. V. Soares, M. C. Calijuri, Deterministic modelling of freshwater lakes and reservoirs: Current trends and recent progress. *Environ. Model. Softw.* **144**, 105143 (2021).

32. E. Deyle, D. Bouffard, V. Frossard, R. Schwefel, J. Melack, G. Sugihara, A hybrid empirical and parametric approach for managing ecosystem complexity: Water quality in Lake Geneva under nonstationary futures. *Proc. Natl. Acad. Sci. U.S.A.* **119**, e2102466119 (2022).

33. B. Fernández Castro, H. E. Chmiel, C. Minaudo, S. Krishna, P. Perolo, S. Rasconi, A. Wüest, Primary and net ecosystem production in a large lake diagnosed from high-resolution oxygen measurements. *Water Resour. Res.* **57**, e2020WR029283 (2021).

34. T. Soomets, T. Kutser, A. Wüest, D. Bouffard, Spatial and temporal changes of primary production in a deep peri-alpine lake. *Inland Waters* **9**, 49–60 (2019).

35. R. Wanninkhof, M. Knox, Chemical enhancement of CO_2 exchange in natural waters. *Limnol. Oceanogr.* **41**, 689–697 (1996).

36. H. Tian, Y. Yao, Y. Li, H. Shi, S. Pan, R. G. Najjar, N. Pan, Z. Bian, P. Ciais, W.-J. Cai, M. Dai, M. A. M. Friedrichs, H.-Y. Li, S. Lohrenz, L. R. Leung, Increased terrestrial carbon export and CO_2 evasion from global inland waters since the preindustrial era. *Global Biogeochem. Cycles* **37**, e2023GB007776 (2023).

37. W. J. van Hoek, J. Wang, L. Vilmin, A. H. W. Beusen, J. M. Mogollón, G. Müller, P. A. Pika, X. Liu, J. J. Langeveld, A. F. Bouwman, J. J. Middelburg, Exploring spatially explicit changes in carbon budgets of global river basins during the 20th century. *Environ. Sci. Technol.* **55**, 16757–16769 (2021).

38. J. P. Casas-Ruiz, P. Bodmer, K. A. Bona, D. Butman, M. Couturier, E. J. S. Emilson, K. Finlay, H. Genet, D. Hayes, J. Karlsson, D. Paré, C. Peng, R. Striegl, J. Webb, X. Wei, S. E. Ziegler, P. A. del Giorgio, Integrating terrestrial and aquatic ecosystems to constrain estimates of land-atmosphere carbon exchange. *Nat. Commun.* **14**, 1571 (2023).

39. G. Abril, A. V. Borges, Ideas and perspectives: Carbon leaks from flooded land: Do we need to replumb the inland water active pipe? *Biogeosciences* **16**, 769–784 (2019).

40. A. Gaudard, R. Schwefel, L. Vinnå, M. Schmid, A. Wüest, D. Bouffard, Optimizing the parameterization of deep mixing and internal seiches in one-dimensional hydrodynamic models: A case study with Simstrat v1.3. *Geosci. Model Dev.* **10**, 3411–3423 (2017).

41. M. Perroud, S. Goyette, A. Martynov, M. Beniston, O. Annevillec, Simulation of multiannual thermal profiles in deep Lake Geneva: A comparison of one-dimensional lake models. *Limnol. Oceanogr.* **54**, 1574–1594 (2009).

42. V. Stepanenko, K. D. Jöhnk, E. Machulskaya, M. Perroud, Z. Subin, A. Nordbo, I. Mammarella, D. Mironov, Simulation of surface energy fluxes and stratification of a small boreal lake by a set of one-dimensional models. *Tellus A Dyn. Meteorol. Oceanogr.* **66**, 21389 (2022).

43. F. Peeters, D. M. Livingstone, G.-H. Goudsmit, R. Kipfer, R. Forster, Modeling 50 years of historical temperature profiles in a large central European lake. *Limnol. Oceanogr.* **47**, 186–197 (2002).

44. G. Fink, M. Schmid, A. Wüest, Large lakes as sources and sinks of anthropogenic heat: Capacities and limits. *Water Resour. Res.* **50**, 7285–7301 (2014).

45. A. Gaudard, L. Råman Vinnå, F. Bärenbold, M. Schmid, D. Bouffard, Toward an open access to high-frequency lake modeling and statistics data for scientists and practitioners–the case of Swiss lakes using Simstrat v2.1. *Geosci. Model Dev.* **12**, 3955–3974 (2019).

46. M. R. Hipsey, Modelling aquatic eco-dynamics: Overview of the AED modular simulation platform, version v0.9.0, Zenodo (2022); https://doi.org/10.5281/zenodo.6516222.

47. R. Schwefel, T. Steinsberger, D. Bouffard, L. D. Bryant, B. Müller, A. Wüest, Using small-scale measurements to estimate hypolimnetic oxygen depletion in a deep lake. *Limnol. Oceanogr.* **63**, S54–S67 (2018).

48. T. Steinsberger, R. Schwefel, A. Wüest, B. Müller, Hypolimnetic oxygen depletion rates in deep lakes: Effects of trophic state and organic matter accumulation. *Limnol. Oceanogr.* **65**, 3128–3138 (2020).

49. A. C. Redfield, The biological control of chemical factors in the environment. *Am. Sci.* **46**, 230A–2221A (1958).

50. P. Perolo, B. Fernández Castro, N. Escoffier, T. Lambert, D. Bouffard, M. E. Perga, Accounting for surface waves improves gas flux estimation at high wind speed in a large lake. *Earth Syst. Dynam.* **12**, 1169–1189 (2021).

51. B. Müller, L. D. Bryant, A. Matzinger, A. Wüest, Hypolimnetic oxygen depletion in eutrophic lakes. *Environ. Sci. Technol.* **46**, 9964–9971 (2012).

52. L. C. Bruce, D. Hamilton, J. Imberger, G. Gal, M. Gophen, T. Zohary, K. D. Hambright, A numerical simulation of the role of zooplankton in C, N and P cycling in Lake Kinneret, Israel. *Ecol. Model.* **193**, 412–436 (2006).

53. G.-Y. Rhee, I. J. Gotham, The effect of environmental factors on phytoplankton growth: Light and the interactions of light with nitrate limitation1. *Limnol. Oceanogr.* **26**, 649–659 (1981).

54. S. van Heuven, D. Pierrot, J. Rae, E. Lewis, D. W. R. Wallace, *CO2SYS v 1.1, MATLAB Program Developed for CO2 System Calculations* (ORNL/CDIAC-105b, Carbon Dioxide Information Analysis Center, Oak Ridge National Laboratory, U.S. DoE, 2011).

55. F. J. Millero, The thermodynamics of the carbonate system in seawater. *Geochim. Cosmochim. Acta* **43**, 1651–1661 (1979).

56. B. Müller, J. S. Meyer, R. Gächter, Alkalinity regulation in calcium carbonate-buffered lakes. *Limnol. Oceanogr.* **61**, 341–352 (2016).

57. L. N. Plummer, E. Busenberg, The solubilities of calcite, aragonite and vaterite in CO_2-H_2O solutions between 0 and 90°C, and an evaluation of the aqueous model for the system $CaCO_3$-CO_2-H_2O. *Geochim. Cosmochim. Acta* **46**, 1011–1040 (1982).

58. A. Groleau, G. Sarazin, B. Vinçon-Leite, B. Tassin, C. Quiblier-Llobéras, Tracing calcite precipitation with specific conductance in a hard water alpine lake (Lake Bourget). *Water Res.* **34**, 4151–4160 (2000).

59. G. Many, N. Escoffier, M. Ferrari, P. Jacquet, D. Odermatt, G. Mariethoz, P. Perolo, M.-E. Perga, Long-Term spatiotemporal variability of whitings in Lake Geneva from multispectral remote sensing and machine learning. *Remote Sens. (Basel)* **14**, 6175 (2022).

60. H. Jansen, R. E. Zeebe, D. A. Wolf-Gladrow, Modeling the dissolution of settling $CaCO_3$ in the ocean. *Global Biogeochem. Cycles* **16**, 11-1–11-16 (2002).

61. Federal Office for the Environment (2022); www.bafu.admin.ch/bafu/en/home/topics/water/state/data/obtaining-monitoring-data-on-the-topic-of-water.html.

62. R. Schwefel, A. Gaudard, A. Wüest, D. Bouffard, Effects of climate change on deepwater oxygen and winter mixing in a deep lake (Lake Geneva): Comparing observational findings and modeling. *Water Resour. Res.* **52**, 8811–8826 (2016).
